# Electronic health record phenotype choice and real-world outcome estimates: A propensity score–matched comparison of three invasive breast cancer definitions in TriNetX

**DOI:** 10.1371/journal.pone.0361883

**Published:** 2026-10-30

**Authors:** Jui-Kun Chiang, Po-Chen Chiang, Malcolm Koo

**Affiliations:** 1 Department of Family Medicine, Dalin Tzu Chi Hospital, Buddhist Tzu Chi Medical Foundation, Dalin, Chiayi, Taiwan; 2 Department of Medical Education, National Taiwan University Hospital, Taipei City, Taiwan; 3 Department of Medical Research, Dalin Tzu Chi Hospital, Buddhist Tzu Chi Medical Foundation, Dalin, Chiayi, Taiwan; 4 Dalla Lana School of Public Health, University of Toronto, Toronto, Ontario, Canada; KL University: KL Deemed to be University, INDIA

## Abstract

Electronic health record-based oncology studies depend on computable case definitions, but different operational definitions may identify different patient populations and change downstream estimates. This retrospective benchmarking cohort study evaluated three definitions of incident invasive breast cancer in the TriNetX Global Collaborative Network: at least one malignant breast cancer diagnosis code (Dx1), a repeated diagnosis-code definition within 90 days (Dx2), and a diagnosis code plus breast cancer-directed treatment evidence (DxTx). Female patients aged 20 years or older were identified after a 12-month washout period and exclusion of patients with carcinoma in situ only. Pairwise one-to-one propensity-score matching was performed using 13 baseline covariates. Outcomes were all-cause mortality and time to the first emergency department visits, assessed from day 180 after the index date to 1, 3, and 5 years after the original index date. Appendicitis and femoral neck fracture were included as negative-control outcomes. Dx1, Dx2, and DxTx identified 1,450,233, 678,739, and 578,576 patients, respectively. After matching, more restrictive definitions were consistently associated with higher hazards of mortality and emergency department visits. For mortality, hazard ratios ranged from 1.26 to 1.32 for DxTx versus Dx1, 1.13 to 1.14 for Dx2 versus Dx1, and 1.13 to 1.17 for DxTx versus Dx2 across assessment windows. Emergency department visit estimates showed a similar but attenuating pattern. These associations should be interpreted as differences between phenotype-defined populations arising from selection and ascertainment mechanisms rather than as treatment effects. Negative-control outcomes were generally close to the null; small appendicitis associations observed in comparisons involving Dx1 did not remain statistically significant after correction for multiple testing. These findings indicate that plausible electronic health record-based breast cancer definitions are not analytically interchangeable. Phenotype specification should therefore be treated as a core analytic decision and evaluated through transparent reporting and sensitivity analyses.

## Introduction

Electronic health records (EHRs) have become an important source of real-world data for oncology research, supporting studies of disease epidemiology, treatment patterns, comparative effectiveness, and health care utilization [1,2]. However, EHRs are generated primarily for clinical care, administration, and billing rather than research. Identifying a cancer cohort from structured EHR data therefore requires a computable phenotype: a rule that translates the clinical construct of interest into specific data elements, codes, time windows, and logical criteria [3]. The choice of phenotype determines who is included, how the index date is assigned, which patients are eligible for matching, and how downstream outcome estimates are generated.

Invasive breast cancer is a useful condition in which to examine these issues. It is one of the most frequently studied cancers in EHR-based research and remains a major global health burden [4,5]. At the same time, EHR-based identification of breast cancer must address several clinically meaningful distinctions, including invasive cancer versus carcinoma in situ, incident versus prevalent disease, and confirmed diagnoses versus suspected or rule-out diagnoses. Small differences in coding logic may therefore produce meaningfully different cohorts.

Operational definitions of breast cancer in EHR-based studies range from minimal diagnosis-code rules to definitions that combine diagnosis codes with treatment evidence [6,7]. A broad single-code definition is expected to improve sensitivity but may retain rule-out diagnoses or miscoded entries. A repeated-code definition, requiring a second confirmatory diagnosis code within a specified interval, is expected to improve specificity but may exclude patients with limited follow-up, fragmented care, or incomplete capture of subsequent encounters. A diagnosis-plus-treatment definition may improve clinical plausibility by requiring evidence of cancer-directed treatment, but it may also select patients with more complete treatment capture, more intensive care pathways, or different disease severity. In a Japanese claims-based validation study, the positive predictive value for breast cancer differed substantially between prevalent-case and incident-case algorithms, from 86.4% to 59.9%, illustrating how design choices in claims-based cancer identification can materially change algorithm performance [8].

Federated EHR research platforms such as the TriNetX Global Collaborative Network make these choices particularly consequential. Large multisite EHR networks allow rapid construction of very large cohorts, but large sample sizes also make small definitional differences statistically detectable. In addition, heterogeneity in coding practices, encounter capture, and treatment recording across health care organizations may affect how an operational definition performs across settings. Despite this, EHR-based breast cancer studies often apply a single case definition without examining how alternative specifications change cohort composition or downstream effect estimates. Negative-control outcomes, which are not expected to be causally affected by the exposure or classification strategy under study, provide one approach for assessing whether observed differences may reflect residual imbalance, selection, or other sources of bias not captured by measured covariates [9].

Rather than establishing diagnostic validity against a clinical gold standard, this study benchmarked the analytic consequences of three plausible EHR-based operational definitions of incident invasive breast cancer spanning increasing levels of diagnostic and treatment confirmation: a single diagnosis-code definition (Dx1), a repeated diagnosis-code definition (Dx2), and a diagnosis code combined with treatment evidence (DxTx). Using the TriNetX Global Collaborative Network [10] with one-to-one propensity score matching on 13 baseline covariates, we compared cohort yield, baseline characteristics, all-cause mortality, and emergency department visits across definitions. Appendicitis and femoral neck fracture were included as negative-control outcomes.

## Methods

### Study design and data source

This retrospective benchmarking cohort study evaluated whether alternative diagnosis-based operational definitions of invasive breast cancer yielded different downstream outcome estimates in real-world electronic health record data. De-identified data were queried and aggregate analysis results were extracted from the TriNetX Global Collaborative Network (TriNetX, LLC) on May 3, 2026. All analyses were performed on the same date. Accordingly, all cohort counts, matching results, and outcome estimates reflect the data available in the network on that date.

TriNetX is a federated research platform that provides access to aggregated electronic health record data from participating health care organizations across inpatient and outpatient care settings. Available data included demographics; diagnoses coded using the International Classification of Diseases, 10th Revision, Clinical Modification (ICD-10-CM); procedures coded using the International Classification of Diseases, 10th Revision, Procedure Coding System (ICD-10-PCS) and/or Current Procedural Terminology (CPT), medications; laboratory measurements mapped to Logical Observation Identifiers Names and Codes (LOINC); and health care utilization records.

### Study population

The source population comprised female patients aged 20 years or older in the TriNetX Global Collaborative Network. Male patients and patients younger than 20 years were excluded. We used an incident invasive breast cancer-oriented design. Invasive breast cancer was identified using ICD-10-CM codes for malignant neoplasm of the breast (C50.*), and codes for carcinoma in situ of the breast (D05.*) were excluded to avoid inclusion of non-invasive disease. Eligible patients were required to have at least 12 months of observable history before cohort entry; this requirement was applied to all patients without exception.

### Washout period and index date

A 12-month washout period before the index date was applied for all case definitions. During this period, patients were required to have no recorded C50.* diagnosis code. Patients with D05.* codes only were not considered to meet the invasive breast cancer definition. The index date was defined separately for each operational definition, as described below.

### Case definitions

Three alternative operational definitions of invasive breast cancer were selected a priori to evaluate how different requirements for diagnosis and treatment evidence influence cohort composition and downstream outcome estimates. The single-diagnosis-code definition (Dx1) required at least one ICD-10-CM C50.* diagnosis code. The repeated-diagnosis-code definition (Dx2) required at least two C50.* diagnosis records, with a repeat diagnosis recorded within 90 days of the initial qualifying diagnosis. The diagnosis-plus-treatment definition (DxTx) required at least one C50.* diagnosis code together with at least one qualifying breast cancer-directed treatment: surgery, chemotherapy, or breast cancer-related medication within 90 days after the index date, or radiotherapy within 180 days after the index date. Dx2 and DxTx represented distinct ascertainment strategies based on repeated diagnosis coding and treatment capture, respectively. These additional requirements were not assumed to confer greater diagnostic validity.

For all three definitions, invasive breast cancer was identified using ICD-10-CM C50.* codes, and D05.* codes were excluded to avoid inclusion of carcinoma in situ only. Pathology findings, tumor stage, tumor grade, and receptor-status criteria were not used to determine cohort membership. The complete diagnosis, procedure, and medication concepts used in the operational definitions are provided in S4 Table.

### Covariates and propensity score matching

Propensity score matching was performed separately for three pairwise comparisons: DxTx versus Dx1, Dx2 versus Dx1, and DxTx versus Dx2. The same 13 prespecified baseline covariate terms were included in each propensity score model: age at index; five race categories recorded in TriNetX (White, Black or African American, Asian, other race, and unknown race); hypertension; diabetes mellitus; stroke; dyslipidemia; chronic kidney disease; body mass index; and serum albumin. The race categories were predefined by TriNetX and were not reclassified by the investigators.

For each comparison, patients from the two phenotype-defined cohorts were matched one-to-one on their propensity scores using the TriNetX built-in matching function, which employed a greedy nearest-neighbor algorithm with a caliper width of 0.1 pooled standard deviations. Each comparison therefore generated its own matched sample, which was used for the corresponding outcome analyses. Covariate balance before and after matching was assessed using absolute standardized mean differences, with values below 0.10 indicating adequate balance on the measured covariates. The underlying platform implementation is proprietary, and individual patient-level matching assignments could not be independently verified by the authors.

### Outcomes and follow-up

The primary clinical outcomes were all-cause mortality and emergency department use. Mortality was evaluated as time to recorded death from any cause; cause-specific mortality, cancer progression, cancer recurrence, progression-free survival, and hospitalization were not evaluated. Emergency department use was evaluated as time to the first recorded emergency department encounter within each assessment window and therefore did not measure the total number of recurrent emergency department visits.

Outcomes were assessed across three prespecified windows: day 180–1 year, day 180–3 years, and day 180–5 years after the original index date. Follow-up began 180 days after the index date to account for the treatment-ascertainment period used in the DxTx definition. The exact TriNetX concepts and coding criteria used to define all clinical and negative-control outcomes are provided in S5 Table.

### Negative-control outcomes

Appendicitis and femoral neck fracture were selected as negative-control outcomes using the diagnosis concepts specified in S5 Table. Each was analyzed as time to the first recorded occurrence within the corresponding assessment window. These outcomes were used to assess whether the alternative case definitions were associated with outcomes that were not expected to be directly determined by the breast cancer phenotype specification after propensity score matching.

### Ethical considerations

The study protocol was reviewed and approved by the Institutional Review Board of the Research Ethics Committee of Buddhist Dalin Tzu Chi Hospital, Taiwan (No. B11402057).

### Statistical analysis

Patient characteristics were summarized descriptively for each operational definition using counts and percentages for categorical variables and summary statistics for continuous variables, where available. Baseline differences before and after matching were evaluated using standardized mean differences. No formal sample size calculation was performed because this was a retrospective analysis of all eligible patients meeting the predefined case definitions in TriNetX.

Within each matched pairwise comparison, all-cause mortality, emergency department visits, appendicitis, and femoral neck fracture were analyzed as time-to-event outcomes. Because the negative-control analyses comprised 18 tests across two outcomes, three pairwise phenotype comparisons, and three assessment windows, p values for these analyses were additionally adjusted using the Holm procedure to control the family-wise error rate. Both nominal and Holm-adjusted p values were considered when interpreting the negative-control findings.

Kaplan-Meier methods were used to estimate event-free probabilities for each follow-up window, and hazard ratios (HRs) with 95% confidence intervals (CIs) were estimated to compare outcomes between definitions. Log-rank tests were used to compare event-free curves between groups. In each pairwise comparison, the first-named definition was treated as the comparison group and the second-named definition as the reference group.

E-values were calculated for the point estimate and for the confidence-limit bound closest to the null to assess the minimum strength of association that an unmeasured confounder would need to have with both the case-definition group and the outcome, conditional on the measured covariates, to fully explain the observed association [11]. A two-sided p value < 0.05 was considered statistically significant. Event-free probabilities were exported from TriNetX and plotted using R (version 4.5.3).

## Results

### Cohort assembly

As of the May 3, 2026 query date, the TriNetX Global Collaborative Network included 82,364,945 female patients aged 20 years or older, who constituted the source population for the three operational definitions. After application of the 12-month washout period and exclusion of patients with carcinoma in situ only, the Dx1, Dx2, and DxTx definitions identified 1,450,233, 678,739, and 578,576 patients, respectively. As expected from the increasing confirmation requirements of the three definitions, the DxTx cohort was the smallest and the Dx1 cohort was the largest. The selection of patients meeting each operational definition is shown in Fig 1.

**Fig 1:**
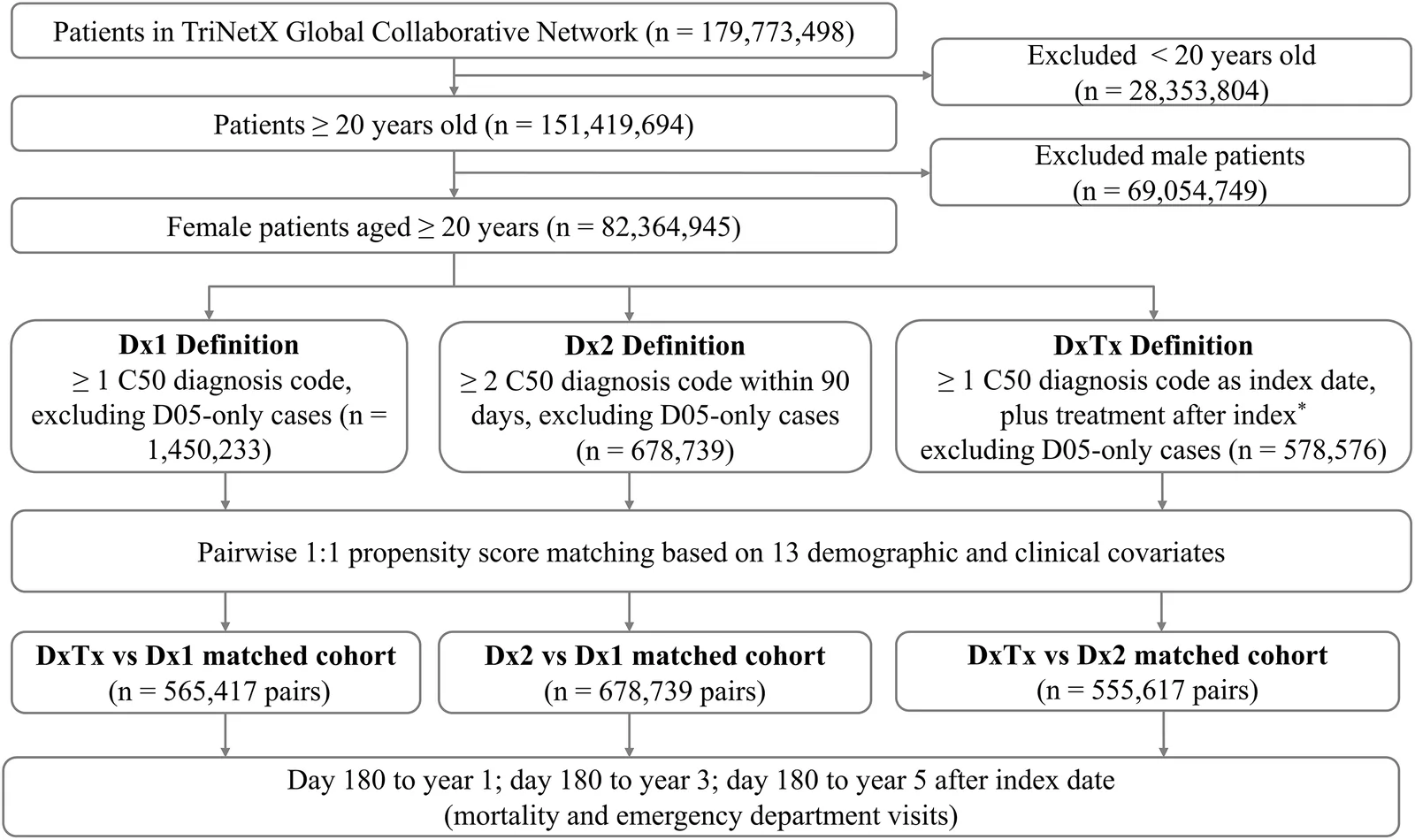
Cohort assembly for three operational definitions of incident invasive breast cancer in TriNetX. Qualifying treatment evidence comprised at least one of the following: surgery, chemotherapy, or breast cancer-related medication within 90 days after the index date, or radiotherapy within 180 days after the index date.

### Baseline characteristics and propensity score matching

Before matching, the three case-definition groups differed on several baseline demographic and clinical variables. After one-to-one propensity score matching on the 13 prespecified baseline covariates, standardized mean differences for all covariates were below the prespecified 0.10 threshold in every pairwise comparison, indicating adequate balance. Matching produced 565,417 pairs for the DxTx versus Dx1 comparison, 678,739 pairs for the Dx2 versus Dx1 comparison, and 555,617 pairs for the DxTx versus Dx2 comparison. Detailed baseline characteristics before and after matching are provided in S1–S3 Tables, and covariate balance before and after matching is summarized visually using Love plots in Fig 2.

**Fig 2:**
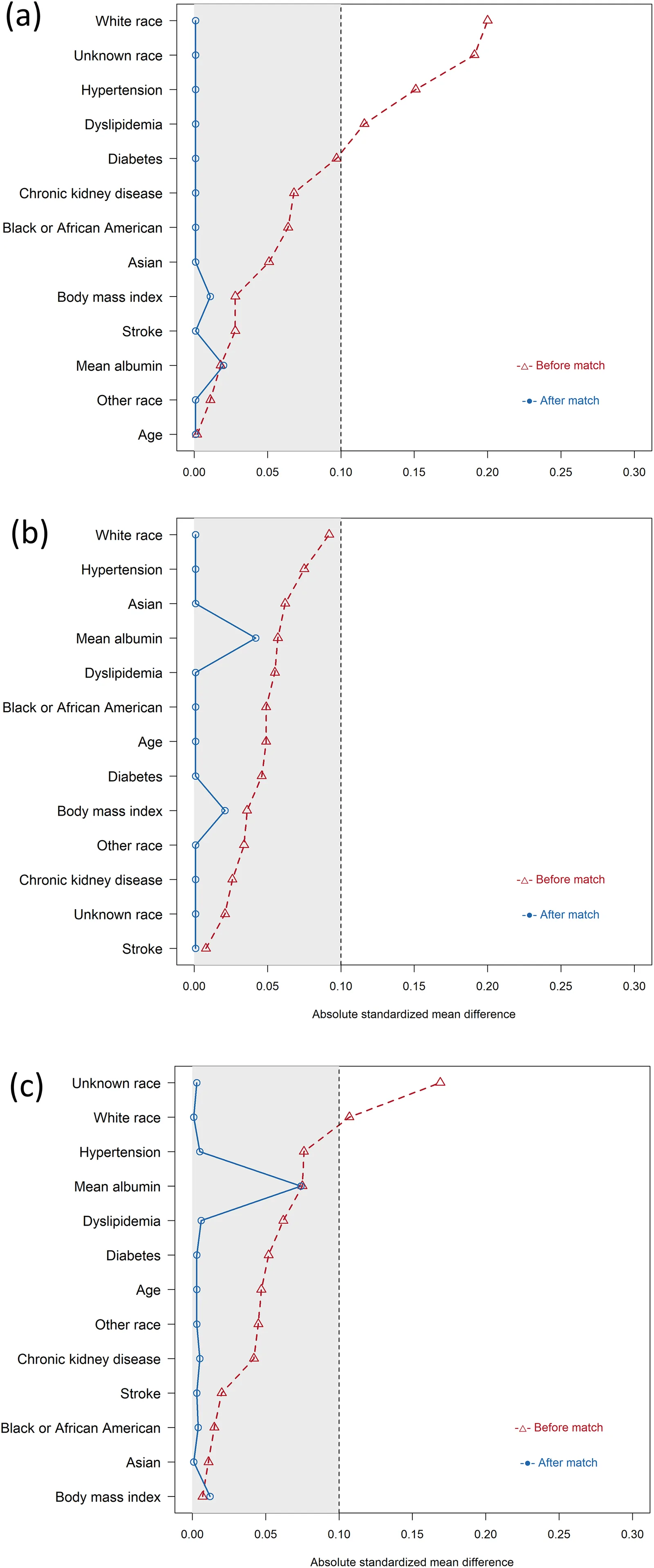
Love plots of covariate balance before and after propensity-score matching across three pairwise comparisons. Absolute standardized mean differences are shown for (a) DxTx versus Dx1, (b) Dx2 versus Dx1, and (c) DxTx versus Dx2. The vertical dashed line indicates the 0.10 threshold for acceptable covariate balance. Dx1, single-diagnosis-code definition; Dx2, repeated-diagnosis-code definition; DxTx, diagnosis-plus-treatment definition.

### All-cause mortality

Higher mortality was observed in the more stringent definition group in every pairwise comparison and across all follow-up windows (Table 1). In the DxTx versus Dx1 comparison, hazard ratios were 1.32 (95% CI 1.27–1.36), 1.29 (1.27–1.31), and 1.26 (1.24–1.28) for the windows from day 180–1, 3, and 5 years after the index date, respectively. The Dx2 versus Dx1 comparison produced smaller and stable hazard ratios of 1.14 (1.10–1.17), 1.14 (1.12–1.16), and 1.13 (1.12–1.15) across the same three windows. The DxTx versus Dx2 comparison fell between these, with hazard ratios of 1.17 (1.14–1.21), 1.14 (1.12–1.16), and 1.13 (1.11–1.14). Hazard ratios attenuated over longer follow-up in the DxTx-involving comparisons but remained stable in the Dx2 versus Dx1 comparison. All log-rank tests reached p < 0.001. E-values for the point estimates ranged from 1.51 to 1.97, and the corresponding E-values for the lower confidence-limit bounds ranged from 1.43 to 1.86.

**Table 1:** All-cause mortality across alternative operational definitions of incident invasive breast cancer after propensity-score matching.

Comparison	Window	Deaths, n, comparison/reference	HR (95% CI)	E-value (lower CI)	p
DxTx vs Dx1 (565,417 pairs)	Day 180–1 year	9,031 / 6,641	1.32 (1.27–1.36)	1.97 (1.86)	<0.001
	Day 180–3 years	32,891 / 25,383	1.29 (1.27–1.31)	1.90 (1.86)	<0.001
	Day 180–5 years	46,271 / 37,311	1.26 (1.24–1.28)	1.83 (1.79)	<0.001
Dx2 vs Dx1 (678,739 pairs)	Day 180–1 year	8,848 / 7,471	1.14 (1.10–1.17)	1.54 (1.43)	<0.001
	Day 180–3 years	33,675 / 28,806	1.14 (1.12–1.16)	1.54 (1.49)	<0.001
	Day 180–5 years	48,375 / 42,307	1.13 (1.12–1.15)	1.51 (1.49)	<0.001
DxTx vs Dx2 (555,617 pairs)	Day 180–1 year	8,809 / 7,499	1.17 (1.14–1.21)	1.62 (1.54)	<0.001
	Day 180–3 years	32,135 / 28,460	1.14 (1.12–1.16)	1.54 (1.49)	<0.001
	Day 180–5 years	45,231 / 40,869	1.13 (1.11–1.14)	1.51 (1.46)	<0.001

Each assessment window began 180 days after the index date and ended at 1, 3, or 5 years after the original index date. Deaths are shown as comparison group/reference group. In each comparison, the first-named definition is the comparison group and the second-named definition is the reference group. E-values are presented for the point estimate, with the E-value for the lower confidence-limit bound in parentheses. CI, confidence interval; Dx1, single-diagnosis-code definition; Dx2, repeated-diagnosis-code definition; DxTx, diagnosis-plus-treatment definition; HR, hazard ratio.

### First recorded emergency department visit

Emergency department visits followed a similar pattern (Table 2). In the DxTx versus Dx1 comparison, hazard ratios were 1.30 (1.28–1.32), 1.22 (1.21–1.23), and 1.20 (1.19–1.21) across the 1-, 3-, and 5-year windows. The Dx2 versus Dx1 hazard ratios were 1.19 (1.18–1.21), 1.15 (1.14–1.16), and 1.14 (1.13–1.15). The DxTx versus Dx2 hazard ratios were smaller, at 1.11 (1.10–1.13), 1.08 (1.07–1.09), and 1.06 (1.06–1.07). Hazard ratios attenuated over longer follow-up in all three pairwise comparisons. All log-rank tests reached p < 0.001. E-values for the point estimates ranged from 1.31 to 1.92.

**Table 2:** Time to the first recorded emergency department visit across alternative operational definitions of incident invasive breast cancer after propensity-score matching.

Comparison	Window	Patients with at least one ED visit, n,comparison/reference	HR (95% CI)	E-value (lower CI)	p
DxTx vs Dx1 (565,417 pairs)	Day 180–1 year	47,037 / 35,410	1.30 (1.28–1.32)	1.92 (1.88)	<0.001
	Day 180–3 years	105,376 / 86,850	1.22 (1.21–1.23)	1.74 (1.71)	<0.001
	Day 180–5 years	127,367 / 108,278	1.20 (1.19–1.21)	1.69 (1.67)	<0.001
Dx2 vs Dx1 (678,739 pairs)	Day 180–1 year	48,834 / 39,662	1.19 (1.18–1.21)	1.67 (1.64)	<0.001
	Day 180–3 years	113,626 / 97,366	1.15 (1.14–1.16)	1.57 (1.54)	<0.001
	Day 180–5 years	140,363 / 122,803	1.14 (1.13–1.15)	1.54 (1.51)	<0.001
DxTx vs Dx2 (555,617 pairs)	Day 180–1 year	45,908 / 41,263	1.11 (1.10–1.13)	1.46 (1.43)	<0.001
	Day 180–3 years	102,886 / 96,650	1.08 (1.07–1.09)	1.37 (1.34)	<0.001
	Day 180–5 years	124,470 / 119,558	1.06 (1.06–1.07)	1.31 (1.31)	<0.001

Each assessment window began 180 days after the index date and ended at 1, 3, or 5 years after the original index date. Counts represent patients with at least one recorded ED visit within each assessment window, shown as comparison group/reference group. Time-to-event analyses used the first recorded ED visit within that window; total visit counts and recurrent ED utilization were not assessed. In each comparison, the first-named definition is the comparison group and the second-named definition is the reference group. E-values are presented for the point estimate, with the E-value for the lower confidence-limit bound in parentheses. CI, confidence interval; Dx1, single-diagnosis-code definition; Dx2, repeated-diagnosis-code definition; DxTx, diagnosis-plus-treatment definition; ED, emergency department; HR, hazard ratio.

### Negative-control outcomes

The negative-control outcomes showed different patterns. For appendicitis (Table 3), the DxTx versus Dx2 comparison showed hazard ratios of 0.93 (0.77–1.12), 1.01 (0.91–1.11), and 0.99 (0.91–1.08) across the three windows, none of which reached statistical significance. The DxTx versus Dx1 and Dx2 versus Dx1 comparisons showed small positive associations at the 3- and 5-year windows, with nominal p values below 0.05. However, none remained statistically significant after Holm adjustment across the 18 negative-control tests; the smallest adjusted p value was 0.126. The DxTx versus Dx2 comparison remained close to the null across all three assessment windows. In the DxTx versus Dx1 comparison, hazard ratios were 1.05 (0.87–1.26) at 1 year, 1.13 (1.02–1.25) at 3 years, and 1.09 (1.00–1.19) at 5 years. The Dx2 versus Dx1 comparison yielded hazard ratios of 1.15 (0.96–1.37), 1.14 (1.03–1.25), and 1.11 (1.03–1.21) across the same windows. E-values for the estimates with nominal p values <0.05 ranged from 1.40 to 1.54, with corresponding lower confidence-limit E-values of 1.00 to 1.21.

**Table 3:** Appendicitis as a negative-control outcome across alternative operational definitions of incident invasive breast cancer after propensity-score matching.

Comparison	Window	Appendicitis events, n,comparison/reference	HR (95% CI)	E-value (lower CI)	Nominal p	Holm-adjusted p
DxTx vs Dx1 (565,417 pairs)	Day 180–1 year	227 / 210	1.05 (0.87–1.26)	1.28 (1.00)	0.649	>0.999
	Day 180–3 years	799 / 702	1.13 (1.02–1.25)	1.51 (1.16)	0.020	0.320
	Day 180–5 years	1,126 / 1,046	1.09 (1.00–1.19)	1.40 (1.00)	0.039	0.585
Dx2 vs Dx1 (678,739 pairs)	Day 180–1 year	278 / 233	1.15 (0.96–1.37)	1.57 (1.00)	0.121	>0.999
	Day 180–3 years	906 / 778	1.14 (1.03–1.25)	1.54 (1.21)	0.009	0.153
	Day 180–5 years	1,312 / 1,164	1.11 (1.03–1.21)	1.46 (1.21)	0.007	0.126
DxTx vs Dx2 (555,617 pairs)	Day 180–1 year	222 / 238	0.93 (0.77–1.12)	1.36 (1.00)	0.433	>0.999
	Day 180–3 years	781 / 781	1.01 (0.91–1.11)	1.11 (1.00)	0.874	>0.999
	Day 180–5 years	1,097 / 1,128	0.99 (0.91–1.08)	1.11 (1.00)	0.831	>0.999

Each assessment window began 180 days after the index date and ended at 1, 3, or 5 years after the original index date. Appendicitis events are shown as comparison group/reference group. In each comparison, the first-named definition is the comparison group and the second-named definition is the reference group. E-values are presented for the point estimate, with the E-value for the confidence-limit bound closest to the null in parentheses. When the confidence interval included the null value, the confidence-limit E-value was 1.00. CI, confidence interval; Dx1, single-diagnosis-code definition; Dx2, repeated-diagnosis-code definition; DxTx, diagnosis-plus-treatment definition; HR, hazard ratio.

For femoral neck fracture (Table 4), all nine hazard ratios across the three pairwise comparisons and three follow-up windows were near unity (range 0.96 to 1.04), and none reached statistical significance. E-values for the point estimates ranged from 1.00 to 1.25. Forest plots summarizing the direction and magnitude of hazard ratios across all clinical and negative-control outcomes for each pairwise comparison are shown in Figs 3–5.

**Table 4:** Femoral neck fracture as a negative-control outcome across alternative operational definitions of incident invasive breast cancer after propensity-score matching.

Comparison	Window	Femoral neck fracture events, n,comparison/reference	HR (95% CI)	E-value (lower CI)	Nominal p	Holm-adjusted p
DxTx vs Dx1 (565,417 pairs)	Day 180–1 year	244 / 229	1.03 (0.86–1.23)	1.21 (1.00)	0.759	>0.999
	Day 180–3 years	714 / 733	0.96 (0.87–1.07)	1.25 (1.00)	0.470	>0.999
	Day 180–5 years	1,022 / 1,048	0.99 (0.91–1.08)	1.11 (1.00)	0.777	>0.999
Dx2 vs Dx1 (678,739 pairs)	Day 180–1 year	248 / 232	1.03 (0.86–1.23)	1.21 (1.00)	0.770	>0.999
	Day 180–3 years	762 / 738	1.01 (0.91–1.11)	1.11 (1.00)	0.914	>0.999
	Day 180–5 years	1,115 / 1,064	1.04 (0.95–1.13)	1.24 (1.00)	0.423	>0.999
DxTx vs Dx2 (555,617 pairs)	Day 180–1 year	235 / 228	1.03 (0.86–1.23)	1.21 (1.00)	0.777	>0.999
	Day 180–3 years	685 / 689	1.00 (0.90–1.11)	1.00 (1.00)	0.986	>0.999
	Day 180–5 years	985 / 1,015	0.99 (0.91–1.08)	1.11 (1.00)	0.798	>0.999

Each assessment window began 180 days after the index date and ended at 1, 3, or 5 years after the original index date. Femoral neck fracture events are shown as comparison group/reference group. In each comparison, the first-named definition is the comparison group and the second-named definition is the reference group. E-values are presented for the point estimate, with the E-value for the confidence-limit bound closest to the null in parentheses. When the confidence interval included the null value, the confidence-limit E-value was 1.00.

CI, confidence interval; Dx1, single-diagnosis-code definition; Dx2, repeated-diagnosis-code definition; DxTx, diagnosis-plus-treatment definition; HR, hazard ratio.

**Fig 3:**
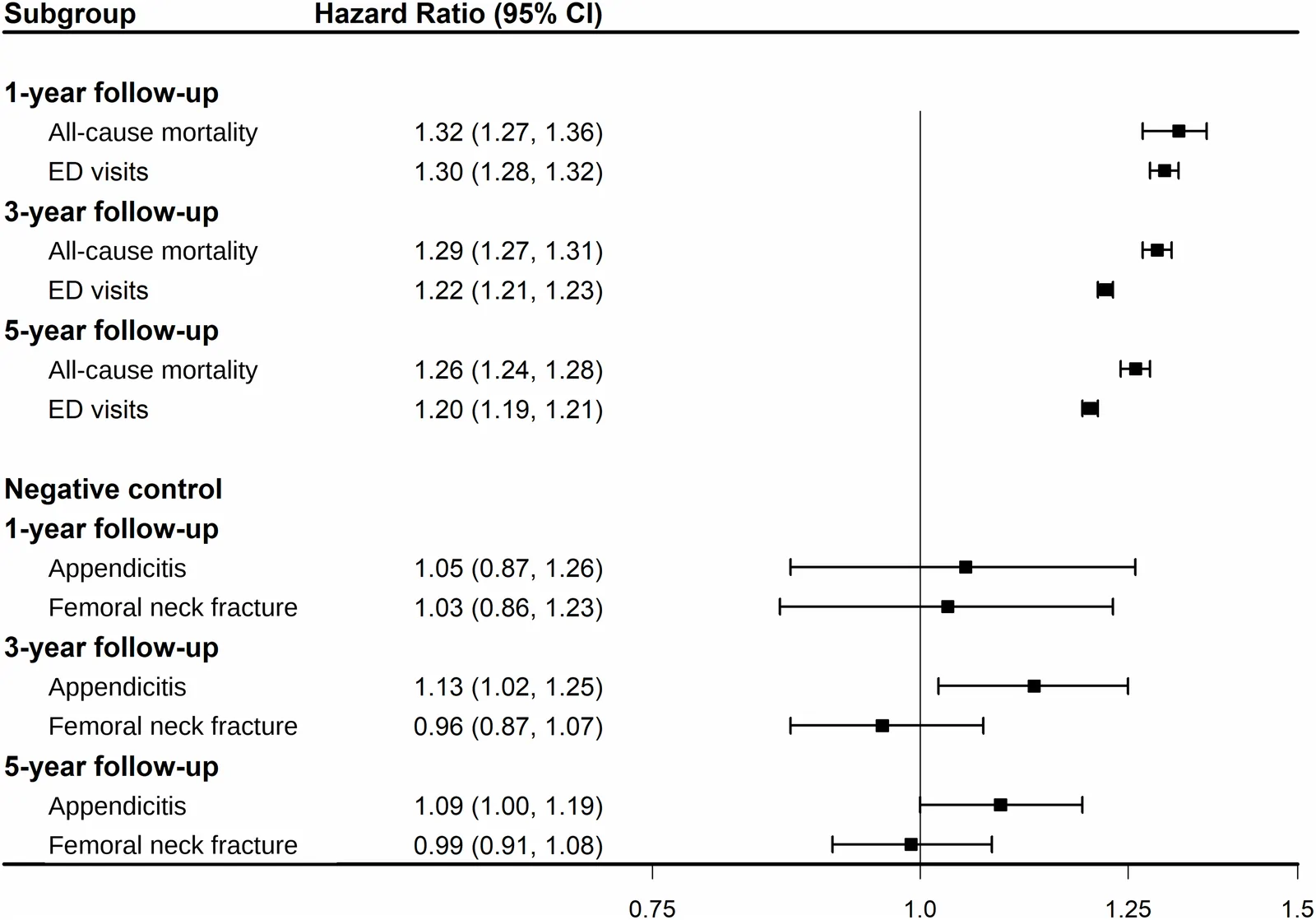
Forest plot of time-to-event associations for DxTx versus Dx1 across clinical and negative-control outcomes. Hazard ratios and 95% confidence intervals are shown for all-cause mortality, first recorded emergency department visits, appendicitis, and femoral neck fracture across the day-180-to-1-year, day-180-to-3-year, and day-180-to-5-year assessment windows. CI, confidence interval; ED, emergency department.

**Fig 4:**
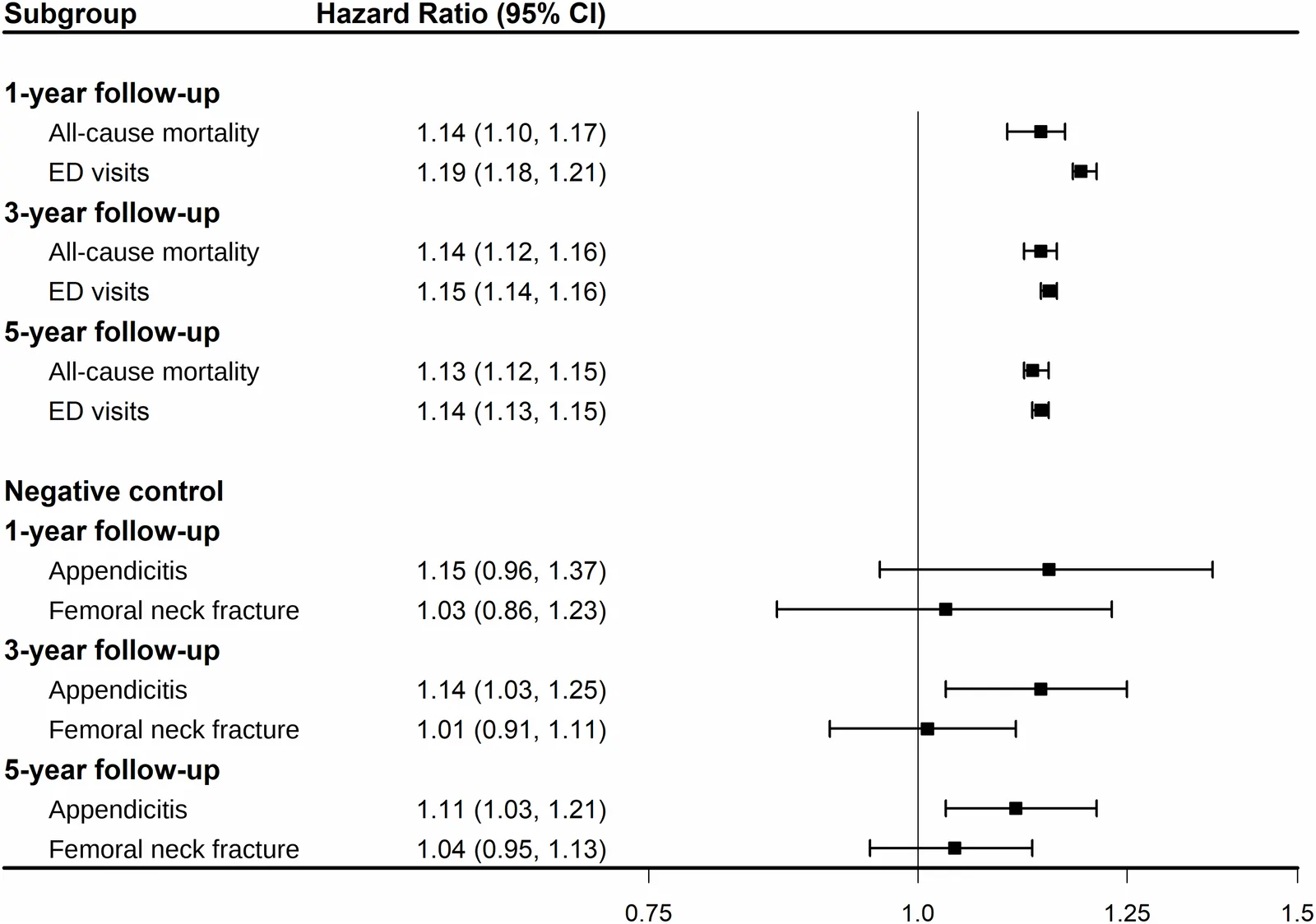
Forest plot of time-to-event associations for Dx2 versus Dx1 across clinical and negative-control outcomes. Hazard ratios and 95% confidence intervals are shown for all-cause mortality, first recorded emergency department visits, appendicitis, and femoral neck fracture across the day-180-to-1-year, day-180-to-3-year, and day-180-to-5-year assessment windows. CI, confidence interval; ED, emergency department.

**Fig 5:**
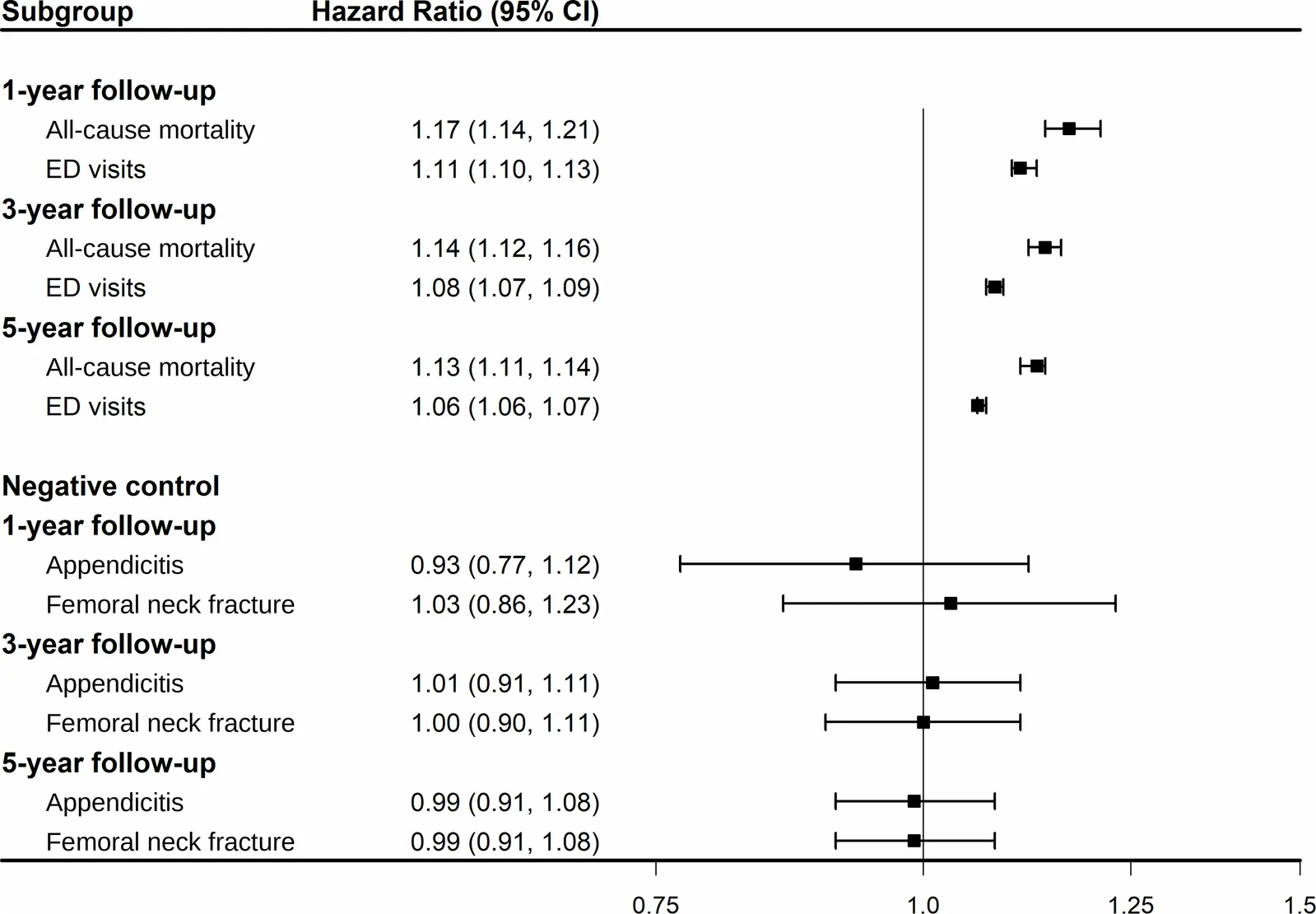
Forest plot of time-to-event associations for DxTx versus Dx2 across clinical and negative-control outcomes. Hazard ratios and 95% confidence intervals are shown for all-cause mortality, first recorded emergency department visits, appendicitis, and femoral neck fracture across the day-180-to-1-year, day-180-to-3-year, and day-180-to-5-year assessment windows. CI, confidence interval; ED, emergency department.

## Discussion

### Principal findings

In this large federated EHR-based benchmarking study, three plausible operational definitions of incident invasive breast cancer produced different cohort yields and downstream outcome estimates. The broad single-code definition (Dx1) identified the largest cohort, whereas the diagnosis-plus-treatment definition (DxTx), which imposed additional treatment-capture requirements, identified the smallest cohort. The cohort-yield pattern is consistent with the additional confirmation requirements imposed by Dx2 and DxTx relative to Dx1, although Dx2 and DxTx represent distinct confirmation strategies based on repeated diagnosis coding and treatment capture, respectively. After pairwise propensity-score matching on 13 baseline covariate terms, definitions requiring additional confirmation were consistently associated with higher hazards of all-cause mortality and the first recorded emergency department visit, with the largest differences observed in comparisons involving DxTx. Importantly, these associations represent differences between phenotype-defined populations and should not be interpreted as adverse effects of breast cancer treatment. Rather, they may reflect a combination of phenotype-related clinical selection, survivor selection, differential observability and ascertainment, and residual differences in unmeasured prognostic characteristics.

The associations persisted across the day-180-to-1-year, day-180-to-3-year, and day-180-to-5-year assessment windows, although hazard ratios generally attenuated over longer follow-up. Negative-control outcomes were generally close to the null. Small positive appendicitis associations were observed at longer follow-up in comparisons involving Dx1, but none remained statistically significant after adjustment for multiple testing; femoral neck fracture remained close to the null across all comparisons and assessment windows. Taken together, these findings indicate that plausible EHR-based breast cancer definitions are not analytically interchangeable and that phenotype specification can materially influence cohort composition and downstream real-world evidence estimates.

### Interpretation of phenotype-dependent differences

The observed gradient across Dx1, Dx2, and DxTx is consistent with the expected trade-off between sensitivity and specificity in EHR-based phenotyping. Dx1, which required at least one C50.* diagnosis code in any encounter setting, likely captured the broadest spectrum of patients, including clinically confirmed cases as well as some patients with rule-out diagnoses, miscoding, limited diagnostic workup, or incomplete longitudinal documentation. Dx2 added a repeated-code requirement within 90 days, providing additional diagnostic confirmation while potentially excluding patients with fragmented care or limited follow-up capture. DxTx was the most restrictive definition because it required both a C50.* diagnosis code and subsequently recorded breast cancer-directed treatment.

DxTx should therefore be viewed as a treatment-capture phenotype rather than simply a more specific diagnostic phenotype. Requiring recorded treatment may select patients whose diagnostic and therapeutic pathway remained visible within participating health care organizations. Two conceptually distinct mechanisms may follow from this requirement. First, clinical selection may enrich DxTx for patients with clinically established disease, greater disease burden, or a need for prompt treatment. Second, differential observability or ascertainment may arise because patients receiving treatment within the network have more health care contacts and therefore more opportunities for subsequent events to be recorded, particularly utilization outcomes such as emergency department visits. This mechanism can increase recorded event rates independently of any difference in the patients’ underlying clinical risk and is therefore conceptually distinct from severity-related selection.

Conversely, DxTx may exclude patients whose treatment occurred outside the network, whose treatment was delayed or less intensive, or whose care was incompletely captured in structured EHR data. These mechanisms provide plausible explanations for why DxTx comparisons showed the largest differences in mortality and emergency department visits despite adequate balance in measured baseline covariates. Accordingly, the higher hazards observed in DxTx comparisons should be interpreted as differences between phenotype-defined populations rather than as treatment effects.

A further consideration is survivor selection introduced by the treatment requirement. Patients must remain alive and observable long enough for qualifying treatment to be recorded within the 90- to 180-day ascertainment period. Considered in isolation, this mechanism would be expected to select a healthier DxTx population and attenuate subsequent mortality. However, an opposing mechanism may operate in the broad Dx1 phenotype. Because Dx1 requires only a single C50.* diagnosis code, it may include patients whose recorded diagnosis did not represent clinically established active invasive breast cancer, including rule-out diagnoses, miscoded entries, or isolated diagnoses without subsequent confirmation. Such patients would be expected, on average, to have lower mortality than patients with clinically established breast cancer. Consistent with this interpretation, Dx2 was also associated with higher mortality than Dx1 despite not requiring treatment confirmation, suggesting that at least part of the mortality gradient may arise from the broad single-code Dx1 phenotype rather than from treatment-based selection alone. The higher post-landmark mortality observed in DxTx should therefore be viewed as the net result of competing selection mechanisms. It does not indicate that survivor selection was absent; rather, its expected downward influence on mortality appears to have been insufficient to offset differences introduced by phenotype definition, disease characteristics, and treatment-pathway selection. Without pathology confirmation, tumor stage, or other detailed prognostic information, the relative contributions of these mechanisms cannot be quantified.

The attenuation of hazard ratios over longer assessment windows is also compatible with a pathway-selection interpretation. Differences between phenotype-defined populations may be greatest during the earlier post-landmark period, when patients captured through treatment-based criteria remain closer to diagnosis and treatment initiation and may experience more intensive care, treatment-related complications, and health care contact. As follow-up lengthened, the contribution of these early pathway differences to the overall contrast diminished, although the associations did not disappear completely.

Finally, the persistence of outcome differences after propensity-score matching suggests that balancing measured baseline covariates does not necessarily make alternative EHR phenotypes clinically equivalent. Matching achieved adequate balance on the 13 prespecified variables. However, these variables do not capture several factors that are highly relevant to breast cancer prognosis and health care utilization, including tumor characteristics, treatment intent, socioeconomic context, insurance status, access to oncology care, and facility-level coding or treatment-recording practices. In addition, the definitions themselves imposed different requirements for diagnostic confirmation, treatment capture, and continued observability. Thus, matched patients could remain systematically different in ways that were not represented in the propensity-score model. Propensity-score matching can reduce imbalance in measured baseline characteristics, but it cannot remove selection or ascertainment mechanisms that arise directly from phenotype construction or from unmeasured clinical features.

### Negative-control outcome analyses

Negative-control outcomes can help detect residual confounding, selection bias, or other systematic errors in observational studies, although their interpretation depends on the plausibility of the assumed absence of a causal relationship with the exposure or classification strategy [12]. In the present study, femoral neck fracture showed hazard ratios close to the null across all pairwise comparisons and follow-up windows. Appendicitis also generally yielded estimates close to the null, although small positive associations were observed at longer follow-up in the DxTx versus Dx1 and Dx2 versus Dx1 comparisons. Four of these appendicitis comparisons had nominal p values below 0.05; however, none remained statistically significant after Holm adjustment across the 18 negative-control tests, with the smallest adjusted p value being 0.126. Thus, the negative-control analyses provided no statistically significant evidence of systematic associations after accounting for multiple testing. The small appendicitis point estimates involving Dx1 may reflect chance variation, although residual differences in health care access, diagnostic capture, or other unmeasured characteristics across phenotype-defined cohorts cannot be excluded.

These findings should not be interpreted as evidence that residual bias was absent. Negative-control outcomes are informative only to the extent that they are unaffected by mechanisms related to cohort definition and outcome ascertainment. Appendicitis may share determinants of health care access, emergency service use, and diagnostic capture with the main utilization outcome. Femoral neck fracture may also be influenced by factors related to breast cancer and its management, including age, frailty, bone health, metastatic disease, and endocrine therapy. The negative-control analyses should therefore be viewed as bias-detection checks rather than validation of the breast cancer phenotypes themselves. In this context, the absence of multiplicity-adjusted associations across the negative-control analyses does not suggest a broad, outcome-independent source of bias affecting all comparisons, but it cannot exclude residual confounding, differential ascertainment, or selection mechanisms specific to the phenotype definitions.

### Relation to previous literature

The present findings are consistent with previous validation and phenotyping studies showing that cancer identification algorithms are sensitive to choices about diagnosis codes, repeated-code requirements, treatment evidence, and washout periods. Prior reviews of administrative and EHR-based breast cancer identification have shown that diagnosis-code algorithms can achieve acceptable performance, but their accuracy varies across databases, coding systems, and algorithm specifications [6]. Similarly, validation studies of claims-based cancer algorithms have reported that different design choices can produce materially different positive predictive values, particularly when distinguishing incident from prevalent disease [7,8]. In breast cancer recurrence research, Xu et al. developed and validated case-finding algorithms using routinely collected administrative data and showed that patterns of health events after initial treatment, including surgery, chemotherapy, radiation, biopsy, and specialist visits, could be used to identify recurrence with varying sensitivity, specificity, and positive predictive value [13]. The present study complements that literature by evaluating how plausible breast cancer definitions behave within the same large federated EHR environment, rather than estimating diagnostic accuracy against chart review or registry data. Its contribution is therefore not to identify a single correct phenotype, but to show that reasonable phenotype choices can alter cohort size, matched comparisons, and downstream outcome estimates.

### Implications for TriNetX and real-world evidence studies

For studies that use TriNetX and other large EHR-based real-world data platforms, the practical implications are direct. Federated EHR networks can support rapid cohort construction and adequately powered analyses, but large sample size does not resolve uncertainty in phenotype specification [14]. Investigators should therefore treat the operational definition as a core analytic decision rather than a minor technical step. The REporting of studies Conducted using Observational Routinely-collected health Data (RECORD) statement extends the Strengthening the Reporting of Observational Studies in Epidemiology (STROBE) statement to studies using routinely collected health data [15]. In line with these guidelines, studies should transparently report the coding logic, data access, and selection processes used to define cohorts. For EHR-based cancer studies, this includes diagnosis codes, encounter settings, washout periods, index-date rules, repeated-code requirements, treatment windows, and exclusion criteria. When feasible, sensitivity analyses using alternative definitions should be conducted to assess whether substantive conclusions are stable across plausible phenotype choices. Broad single-code definitions may be useful for sensitivity-oriented or exploratory analyses, whereas repeated-code or treatment-confirmed definitions may be preferable when diagnostic certainty is prioritized. However, more restrictive definitions can introduce their own selection mechanisms, particularly when they require treatment capture. Phenotype stringency should therefore be chosen according to the research question, data structure, and expected direction of misclassification, rather than assumed to be inherently superior.

### Strengths and limitations

This study has several strengths. It used a large federated EHR network, which allowed direct comparison of alternative breast cancer definitions within the same data environment, and it evaluated three clinically plausible definitions spanning different levels of diagnostic and treatment confirmation rather than a single code-based algorithm. All pairwise comparisons were analyzed after one-to-one propensity-score matching on a prespecified covariate set, with post-matching balance assessed using standardized mean differences, and outcomes were examined across multiple follow-up windows to show whether definitional differences persisted or attenuated. Two negative-control outcomes provided an additional check for residual imbalance, and follow-up began at day 180 after the index date to align outcome ascertainment with completion of the treatment-ascertainment window used in the DxTx definition.

Several limitations should be considered. First, this study did not include chart review, tumor registry linkage, pathology confirmation, or manual adjudication; therefore, the findings should be interpreted as benchmarking analytic consequences rather than validating diagnostic accuracy. Second, misclassification of invasive breast cancer, incident disease status, treatment exposure, and outcomes remains possible because the analysis relied on structured EHR data. Third, DxTx required treatment evidence in addition to a diagnosis code, whereas Dx2 required repeated diagnosis coding. Comparisons involving DxTx may therefore reflect differences in treatment capture, clinical selection, and diagnostic ascertainment. Fourth, tumor stage, receptor status, tumor grade, recurrence status, treatment intent, performance status, prior cancer history, socioeconomic factors, and cause-specific mortality were not available for adjustment in the present analysis and were not included in the propensity score models. These factors influence prognosis and treatment selection, so adequate balance on the measured covariates does not establish comparability in tumor characteristics or disease severity. This limitation is particularly relevant to DxTx because requiring recorded cancer-directed treatment may select patients according to clinical severity and treatment indication. Residual differences in these characteristics may therefore contribute to the observed associations with mortality and the first recorded emergency department visit. Fifth, emergency department use was analyzed as time to the first recorded visit and therefore did not capture recurrent utilization or the total burden of emergency department care. The aggregate TriNetX analytic environment available for this study did not provide the patient-level longitudinal event data required to apply recurrent-event, mean cumulative function, or count-based models. Consequently, the findings should be interpreted as differences in the occurrence and timing of the first recorded emergency department visit rather than differences in total emergency department utilization. Sixth, although propensity-score matching achieved balance on measured baseline covariates, residual confounding and selection bias may persist. Finally, TriNetX data depend on the coding practices, data completeness, and participation patterns of contributing health care organizations, which may limit generalizability to other health systems or data environments.

## Conclusion

This study showed that alternative EHR-based operational definitions of incident invasive breast cancer produced different cohort sizes and downstream outcome estimates in TriNetX, even after propensity-score matching. Definitions with additional confirmation requirements, particularly the diagnosis-plus-treatment definition, were associated with higher hazards of all-cause mortality and the first emergency department visit than broader diagnosis-code definitions. Negative-control outcomes were generally close to the null, and none remained statistically significant after Holm adjustment, although residual confounding, selection, and differential ascertainment cannot be excluded. These findings support transparent reporting, sensitivity analyses, and cautious interpretation when applying diagnosis-based breast cancer phenotypes in real-world data studies.

## Supporting information

S1 Table Baseline characteristics before and after propensity-score matching for DxTx versus Dx1. (DOCX)

S2 Table Baseline characteristics before and after propensity-score matching for Dx2 versus Dx1. (DOCX)

S3 Table Baseline characteristics before and after propensity-score matching for DxTx versus Dx2. (DOCX)

S4 Table Operational definitions and code sets used to identify invasive breast cancer phenotypes in TriNetX. (DOCX)

S5 Table Operational definitions and coding criteria for clinical and negative-control outcomes in TriNetX. (DOCX)
