# Supplementary material for: Electronic health record phenotype choice and real-world outcome estimates: A propensity score–matched comparison of three invasive breast cancer definitions in TriNetX
Source: PLoS One. 2026 Oct 30;21(10):e0361883. doi: 10.1371/journal.pone.0361883 (PMC13722328; doi:10.1371/journal.pone.0361883)
Supplement: Supplementary file 1 [file pone.0361883.s001.docx]

**S1 Table. Baseline characteristics before and after propensity-score matching: DxTx versus Dx1.**

| **Variable** | **Before matching** | | |  | **After matching** | | |
| --- | --- | --- | --- | --- | --- | --- | --- |
|  | **DxTx** (n = 578,576) | **Dx1** (n = 1,450,233) | **SMD** |  | **DxTx** (n = 565,417) | **Dx1** (n = 565,417) | **SMD** |
| Age at index, mean (SD), years | 61.0 (13.6) | 61.1 (13.8) | 0.002 |  | 61.0 (13.6) | 61.0 (13.6) | <0.001 |
| Race |  |  |  |  |  |  |  |
| White | 349,391 (60.4) | 732,674 (50.5) | 0.200 |  | 349,391 (61.8) | 349,393 (61.8) | <0.001 |
| Unknown race | 93,509 (16.2) | 344,456 (23.8) | 0.191 |  | 93,509 (16.5) | 93,509 (16.5) | <0.001 |
| Black or African American | 56,084 (9.7) | 114,354 (7.9) | 0.064 |  | 56,084 (9.9) | 56,084 (9.9) | <0.001 |
| Other race | 32,052 (5.5) | 84,013 (5.8) | 0.011 |  | 32,052 (5.7) | 32,052 (5.7) | <0.001 |
| Asian | 30,093 (5.2) | 59,867 (4.1) | 0.051 |  | 30,096 (5.3) | 30,096 (5.3) | <0.001 |
| Comorbidities |  |  |  |  |  |  |  |
| Hypertension | 92,221 (15.9) | 156,695 (10.8) | 0.151 |  | 92,221 (16.3) | 92,221 (16.3) | <0.001 |
| Diabetes | 39,166 (6.8) | 65,588 (4.5) | 0.097 |  | 39,166 (6.9) | 39,168 (6.9) | <0.001 |
| Stroke | 4,235 (0.7) | 7,450 (0.5) | 0.028 |  | 4,235 (0.7) | 4,235 (0.7) | <0.001 |
| Dyslipidemia | 48,958 (8.5) | 79,842 (5.5) | 0.116 |  | 48,958 (8.7) | 48,956 (8.7) | <0.001 |
| Chronic kidney disease | 16,022 (2.8) | 25,445 (1.8) | 0.068 |  | 16,022 (2.8) | 16,020 (2.8) | <0.001 |
| Body mass index, mean (SD), kg/m^2^ | 29.4 (7.1) | 29.2 (7.1) | 0.028 |  | 29.4 (7.1) | 29.3 (7.1) | 0.011 |
| Albumin, mean (SD), g/dL | 4.0 (0.5) | 4.0 (0.5) | 0.018 |  | 4.0 (0.5) | 4.0 (0.5) | 0.020 |

SD, standard deviation. SMD, standardized mean differences.

Race/ethnicity categories do not sum to 100% because less frequent categories are not displayed and because of rounding. Percentages were calculated using the denominator shown for each cohort.
