# Supplementary material for: Electronic health record phenotype choice and real-world outcome estimates: A propensity score–matched comparison of three invasive breast cancer definitions in TriNetX
Source: PLoS One. 2026 Oct 30;21(10):e0361883. doi: 10.1371/journal.pone.0361883 (PMC13722328; doi:10.1371/journal.pone.0361883)
Supplement: Supplementary file 2 [file pone.0361883.s002.docx]

**S2 Table.** Baseline characteristics before and after propensity-score matching: Dx2 versus Dx1.

| **Variable** | **Before matching** | | |  | **After matching** | | |
| --- | --- | --- | --- | --- | --- | --- | --- |
|  | **Dx2** (n = 678,739) | **Dx1** (n = 1,450,233) | **SMD** |  | **Dx2** (n = 678,739) | **Dx1**  (n = 678,739) | **SMD** |
| Age at index, mean (SD), years | 60.4 (13.5) | 61.1 (13.8) | 0.049 |  | 60.4 (13.5) | 60.4 (13.5) | <0.001 |
| Race |  |  |  |  |  |  |  |
| White | 373,973 (55.1) | 732,674 (50.5) | 0.092 |  | 373,973 (55.1) | 373,976 (55.1) | <0.001 |
| Unknown race | 155,063 (22.8) | 344,456 (23.8) | 0.021 |  | 155,063 (22.8) | 155,063 (22.8) | <0.001 |
| Black or African American | 62,825 (9.3) | 114,354 (7.9) | 0.049 |  | 62,825 (9.3) | 62,825 (9.3) | <0.001 |
| Other race | 44,919 (6.6) | 84,013 (5.8) | 0.034 |  | 44,919 (6.6) | 44,919 (6.6) | <0.001 |
| Asian | 36,953 (5.4) | 59,867 (4.1) | 0.062 |  | 36,953 (5.4) | 36,952 (5.4) | <0.001 |
| Comorbidities |  |  |  |  |  |  |  |
| Hypertension | 89,956 (13.3) | 156,695 (10.8) | 0.075 |  | 89,956 (13.3) | 89,958 (13.3) | <0.001 |
| Diabetes | 37,475 (5.5) | 65,588 (4.5) | 0.046 |  | 37,475 (5.5) | 37,474 (5.5) | <0.001 |
| Stroke | 3,862 (0.6) | 7,450 (0.5) | 0.008 |  | 3,862 (0.6) | 3,864 (0.6) | <0.001 |
| Dyslipidemia | 46,310 (6.8) | 79,842 (5.5) | 0.055 |  | 46,310 (6.8) | 46,309 (6.8) | <0.001 |
| Chronic kidney disease | 14,376 (2.1) | 25,445 (1.8) | 0.026 |  | 14,376 (2.1) | 14,377 (2.1) | <0.001 |
| Body mass index, mean (SD), kg/m^2^ | 29.5 (7.1) | 29.2 (7.1) | 0.036 |  | 29.5 (7.1) | 29.3 (7.1) | 0.021 |
| Albumin, mean (SD), g/dL | 4.0 (0.5) | 4.0 (0.5) | 0.057 |  | 4.0 (0.5) | 4.0 (0.5) | 0.042 |

SD, standard deviation. SMD, standardized mean difference.

Race/ethnicity categories do not sum to 100% because less frequent categories are not displayed and because of rounding. Percentages were calculated using the denominator shown for each cohort.
