# Supplementary material for: Electronic health record phenotype choice and real-world outcome estimates: A propensity score–matched comparison of three invasive breast cancer definitions in TriNetX
Source: PLoS One. 2026 Oct 30;21(10):e0361883. doi: 10.1371/journal.pone.0361883 (PMC13722328; doi:10.1371/journal.pone.0361883)
Supplement: Supplementary file 3 [file pone.0361883.s003.docx]

**S3 Table.** Baseline characteristics before and after propensity-score matching: DxTx versus Dx2.

| **Variable** | **Before matching** | | |  | **After matching** | | |
| --- | --- | --- | --- | --- | --- | --- | --- |
|  | **DxTx** (n = 578,576) | **Dx2** (n = 678,739) | **SMD** |  | **DxTx** (n = 555,617) | **Dx2** (n = 555,617) | **SMD** |
| Age at index, mean (SD), years | 61.0 (13.6) | 60.4 (13.5) | 0.047 |  | **60.9 (13.6)** | 60.9 (13.6) | 0.003 |
| Race |  |  |  |  |  |  |  |
| White | 349,305 (60.4) | 373,886 (55.1) | 0.107 |  | 340,895 (61.4) | 341,065 (61.4) | <0.001 |
| Unknown race | 93,506 (16.2) | 155,068 (22.8) | 0.169 |  | 93,506 (16.8) | 92,949 (16.7) | 0.003 |
| Black or African American | 56,081 (9.7) | 62,818 (9.3) | 0.015 |  | 55,042 (9.9) | 55,714 (10.0) | 0.004 |
| Other race | 32,047 (5.5) | 44,910 (6.6) | 0.045 |  | 32,032 (5.8) | 31,594 (5.7) | 0.003 |
| Asian | 30,093 (5.2) | 36,952 (5.4) | 0.011 |  | 29,902 (5.4) | 30,038 (5.4) | 0.001 |
| Comorbidities |  |  |  |  |  |  |  |
| Hypertension | 92,190 (15.9) | 89,929 (13.3) | 0.076 |  | 86,752 (15.6) | 85,813 (15.4) | 0.005 |
| Diabetes | 39,149 (6.8) | 37,464 (5.5) | 0.052 |  | 36,145 (6.5) | 35,785 (6.4) | 0.003 |
| Stroke | 4,235 (0.7) | 3,861 (0.6) | 0.020 |  | 3,789 (0.7) | 3,631 (0.7) | 0.003 |
| Dyslipidemia | 48,937 (8.5) | 46,291 (6.8) | 0.062 |  | 45,644 (8.2) | 44,781 (8.1) | 0.006 |
| Chronic kidney disease | 16,010 (2.8) | 14,367 (2.1) | 0.042 |  | 14,149 (2.5) | 13,749 (2.5) | 0.005 |
| Body mass index, mean (SD), kg/m^2^ | 29.4 (7.1) | 29.5 (7.1) | 0.007 |  | 29.4 (7.1) | 29.5 (7.1) | 0.012 |
| Albumin, mean (SD), g/dL | 4.0 (0.5) | 4.0 (0.5) | 0.075 |  | 4.0 (0.5) | 4.1 (0.5) | 0.074 |

SD, standard deviation. SMD, standardized mean differences.

Race/ethnicity categories do not sum to 100% because less frequent categories are not displayed and because of rounding. Percentages were calculated using the denominator shown for each cohort.
