# Supplementary material for: Electronic health record phenotype choice and real-world outcome estimates: A propensity score–matched comparison of three invasive breast cancer definitions in TriNetX
Source: PLoS One. 2026 Oct 30;21(10):e0361883. doi: 10.1371/journal.pone.0361883 (PMC13722328; doi:10.1371/journal.pone.0361883)
Supplement: Supplementary file 4 [file pone.0361883.s004.docx]

**S4 Table. Operational definitions and code sets used to identify invasive breast cancer phenotypes in TriNetX.**

| **Phenotype component** | **Dx1** | **Dx2** | **DxTx** | **Ascertainment window** | **Coding system/concepts** |
| --- | --- | --- | --- | --- | --- |
| Malignant breast cancer diagnosis | Required | Required | Required | Index date | ICD-10-CM C50.* |
| Prior malignant breast cancer diagnosis | Excluded | Excluded | Excluded | 12 months before index date | ICD-10-CM C50.* |
| Carcinoma in situ | Did not qualify | Did not qualify | Did not qualify | Cohort identification | ICD-10-CM D05.* |
| Repeat malignant breast cancer diagnosis | No | Required | No | Within 90 days | ICD-10-CM C50.* |
| Surgery | No | No | Qualifying treatment | Within 90 days | CPT 1003143 |
| Chemotherapy | No | No | Qualifying treatment | Within 90 days | TriNetX curated concept: 1002 |
| Radiotherapy | No | No | Qualifying treatment | Within 180 days | SNOMED 1287742003 |
| Breast cancer-related medication | No | No | Qualifying treatment | Within 90 days | Tamoxifen [10324], toremifene [38409], anastrozole [84857], letrozole [72965], exemestane [258494], fulvestrant [282357], goserelin [50610], leuprolide [42375], palbociclib [1601374], everolimus [141704] |
| Pathology | Not used | Not used | Not used | Not applicable | Not applicable |
| Tumor stage/grade | Not used | Not used | Not used | Not applicable | Not applicable |
| Receptor status | Not used | Not used | Not used | Not applicable | Not applicable |
