# Supplementary material for: Electronic health record phenotype choice and real-world outcome estimates: A propensity score–matched comparison of three invasive breast cancer definitions in TriNetX
Source: PLoS One. 2026 Oct 30;21(10):e0361883. doi: 10.1371/journal.pone.0361883 (PMC13722328; doi:10.1371/journal.pone.0361883)
Supplement: Supplementary file 5 [file pone.0361883.s005.docx]

**S5 Table.** **Operational definitions and coding criteria for clinical and negative-control outcomes in TriNetX.**

| **Outcome** | **Role** | **Operational definition** | **Coding system / TriNetX concept** | **Exact code/concept** | **Analysis** |
| --- | --- | --- | --- | --- | --- |
| All-cause mortality | Primary clinical outcome | Recorded death from any cause | TriNetX mortality concept | Deceased | Time to death |
| ED visit | Primary clinical outcome | First recorded ED encounter | TriNetX encounter concept | Visit: emergency | Time to first ED visit |
| Appendicitis | Negative control | First recorded appendicitis diagnosis | ICD-10-CM | K35, K36, K37 | Time to first event |
| Femoral neck fracture | Negative control | First recorded femoral neck fracture diagnosis | ICD-10-CM | S72.009A | Time to first event |

ED, Emergency department; ICD-10-CM, International Classification of Diseases, 10th Revision, Clinical Modification.
